# Advanced Metallic and Polymeric Coatings for Neural Interfacing: Structures, Properties and Tissue Responses

**DOI:** 10.3390/polym13162834

**Published:** 2021-08-23

**Authors:** Pengfei Yin, Yang Liu, Lin Xiao, Chao Zhang

**Affiliations:** Department of Biomedical Engineering, Sun Yat-sen University, Shenzhen 518107, China; yinpf3@mail2.sysu.edu.cn (P.Y.); xiaolin23@mail.sysu.edu.cn (L.X.)

**Keywords:** neural interfacing, polymers, metals, coatings, electrodes, structure-property correlations, biocompatibility

## Abstract

Neural electrodes are essential for nerve signal recording, neurostimulation, neuroprosthetics and neuroregeneration, which are critical for the advancement of brain science and the establishment of the next-generation brain–electronic interface, central nerve system therapeutics and artificial intelligence. However, the existing neural electrodes suffer from drawbacks such as foreign body responses, low sensitivity and limited functionalities. In order to overcome the drawbacks, efforts have been made to create new constructions and configurations of neural electrodes from soft materials, but it is also more practical and economic to improve the functionalities of the existing neural electrodes via surface coatings. In this article, recently reported surface coatings for neural electrodes are carefully categorized and analyzed. The coatings are classified into different categories based on their chemical compositions, i.e., metals, metal oxides, carbons, conducting polymers and hydrogels. The characteristic microstructures, electrochemical properties and fabrication methods of the coatings are comprehensively presented, and their structure–property correlations are discussed. Special focus is given to the biocompatibilities of the coatings, including their foreign-body response, cell affinity, and long-term stability during implantation. This review article can provide useful and sophisticated insights into the functional design, material selection and structural configuration for the next-generation multifunctional coatings of neural electrodes.

## 1. Introduction

**Figure 1:**
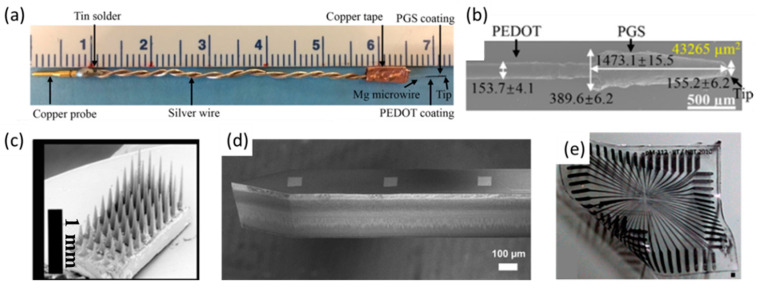
The macroscopic image (**a**) and morphology (**b**) of an Mg-based microwire electrode. An illustration of Utah-type (**c**), Michigan-type (**d**) and PDMS-based electrodes (**e**). (**a**,**b**) are reproduced with permission from [14], Copyright 2020, Elsevier. (**c**) is reproduced with permission from [18], Copyright 2014, iopscience.iop.org. (**d**) is reproduced with permission from [19], Copyright 2015, Elsevier. (**e**) is reproduced with permission from [27], Copyright 2011, Elsevier.

During the last few decades, neural electrodes have been considered as a promising interfacing technology for the direct probing and interfering of the neural tissues [1]. Neural interfaces have been employed to study the basic interactions of the brain–neural system, and to treat many neurophysiologic disorders, such as Parkinson’s disease [2], deafness [3], blindness [4], epilepsy [5] and dyskinesia [6]. They can record a variety of physiologic signals from neural tissues and give stimulations to abnormal neurons to restore the neural system during their implantations. Many neural electrodes have been applied successfully for neural recording and stimulation [7,8,9]. However, several requirements must be met before the neural electrodes can be used in long-term implantation applications. First of all, the materials used to fabricate neural electrodes should be not only properly functioning in vivo but also biocompatible and durable in order to maintain the neural interface. Secondly, the electrical properties of neural electrodes should be engineered to the level suitable for distinct recording and stable stimulation, i.e., low impedance and a high charge injection limit. In order to minimize the damage applied to the brain tissues during implantation, the neural electrodes are generally fabricated into the form of microelectrodes. There are two typical types of microelectrodes, including microwires and micro-electromechanical system (MEMS) arrays. Different kinds of materials have been used to manufacture neural microelectrodes. Noble metals—such as gold [10], platinum [11] and tungsten [12]—are traditional choices for microwire-type microelectrodes. Magnesium, due to its biodegradable property, has also been studied for neural interfacing in order to avoid additional surgery after implantation [13,14] (Figure 1a,b). Conducting materials other than metals—such as carbon nanotubes (CNT) [15], graphene [16] and conducting polymers [17]—have been recently utilized to fabricate MEMS arrays. The microelectrode-based MEMS arrays have been successfully applied in clinical neurophysiologic diagnosis and therapy for a few decades. Typical MEMS arrays include Utah arrays (Figure 1c) [18] and Michigan microelectrodes (Figure 1d) [19].

Noble metals possess excellent electrical conductivity, chemical stability and good biocompatibility, making them suitable for the fabrication of neural electrodes. However, there are certain limitations in metallic electrodes due to the mismatch between rigid metals and soft neural tissues regarding their electrical, mechanical and biological properties, making it hard to realize a metallic neural electrode with high sensitivity and spatial accuracy [20]; precise, tunable stimulation; and the least tissue response [21]. There are two different charge transport carriers in tissue and electrodes, i.e., ions for neural tissues and electrons for electrodes, resulting in electrical imparity [22]. The difference in the mechanical properties may cause a huge disparity in the Young’s modulus between neural tissue (kPa level) and the metallic electrode (GPa level). As the neural electrodes are implanted in brain neural tissues, it would induce damage to neurons and cause blood capillary fracture, resulting in the leakage of the blood—brain barrier and a tissue response [23]. Meanwhile, the implanted electrodes, while recognized as foreign body objects, will be attacked by the immune system, resulting in a series of inflammatory responses and the accumulation of microglia at the implant site, which may eventually cause the isolation of the electrodes from the tissue and the failure of the electrode [24]. The optimal design of an electrode with a conformal geometric size to reduce the implantation damage, the utilization of soft materials with a similar Young’s modulus to the neural tissue are state-of-the-art concepts for the research and development of next-generation neural electrodes [25,26]. As the geometric size of metallic electrodes is reduced, their suitability for neural recordings may be significantly hindered due to their low charge injection capability and charge storage capacitance. A few soft organic polymers (PDMS (Figure 1e) [27,28], PI [29], parylene C [30], PU [31]) have been used to fabricate neural electrodes in order to mitigate the mechanical mismatch in the tissue–electrode interface. Compared to metals and carbon materials, polymeric materials are less efficient for the transfer of the electrical signals due to their low electrical conductivity [32]. The polymeric materials are also difficult to penetrate the body and fix in a specific implant site with due to their low Young’s modulus. In order to prepare neural electrodes with both conformal characteristics and minimal geometric size, nanofabrication methods such as optical lithography have been introduced to fabricate sheet-shape MEMS arrays [33]. However, the intrinsic physical and chemical differences between the electrodes and neuronal tissues would induce an unstable electronic–tissue interface, which can be subsequently targeted and eliminated by the immune system; on the other, the conductive layer alone cannot facilitate advanced functions such as neuro-regeneration, molecular delivery and anti-inflammatory effects. In order to address these issues, functional coatings can be applied on the electrode surface in order to achieve the designated properties, biocompatibility and durability. The benefits of functional coatings may include, but are not limited to: (i) increasing the geometric surface area of the electrode; (ii) enhancing the electrochemical properties of the electrode; (iii) lowering the modulus of the electrode surface; (iv) reducing the foreign body response in the electrode–tissue interface; and (v) integrating advanced therapeutical functions into the electrode. Compared to the manipulation of the structure and composition of the electrode alone, surface coating is a relatively easy and cost-effective methodology to fabricate multiscale and multifunctional hybrid electrodes, while the coating materials and their microstructures can be selected and tuned in a user-specific manner.

Thus, it is crucial to review the current situation of the neural electrode coatings and the recent developments in the coating procedures and technologies in the research field of neural interfacing. The coating on the electrode surface, which can serve as an interlayer between the neural electrodes and the target tissues, plays a significant role in the efficiency of the abiotic/biotic interface. For example, a coating layer with poor biocompatibility and electrical conductivity will significantly prevent the effectiveness of signal transduction in the interface. An interface with unsatisfactory chemical and mechanical properties may cause severe responses in the surrounding tissues, resulting in the failure of the neural device. Motivated by the above-mentioned issues, we undertake this review of coatings applied on the surface of neural electrodes, which highlights various successful works in this emerging field, and the existing challenges in the design of an ideal coating for a neural interface.

In the following sections, the mechanism of neural tissue responses to the implantable electrode in the electrode–tissue interface is discussed first. The materials used for the coating (e.g., metals and their derivatives, carbon materials, conducting polymeric materials, and hydrogel coatings) will be discussed in detail based on their electrical, mechanical and biological properties, and the corresponding fabrication methods. Correlations between the structure and properties of the coating also will be discussed. After all of this, perspectives on the challenges in the field and the future developments of the neural electrode coatings are depicted and demonstrated.

## 2. Tissue Responses to Implantable Neural Electrodes

When the electrodes are implanted in the central nervous system for a long period of time, they may trigger inflammatory responses [34,35]. The cells generated by the inflammatory response may include astrocytes, microglia and oligodendrocytes, etc. All of these cells can secrete inhibitory substances that impede the regeneration of axons. During the implantation of neural electrodes, the blood–brain barrier and blood vessels may be damaged, causing nerve cell death near the surface of the electrode. Then, the microglia and sources of activated macrophages in the blood may begin to migrate to the surface of the neural electrode and release cytokines, chemokines and neurotransmitters, such as oxygen free radicals for the cleaning of the dead neurons to constitute an acute inflammatory response [36]. The presence of implant trauma and an inflammatory response can result in tissue edema [34,37], which may cause the neurons around the electrode to move away from the electrode’s surface. Moreover, as the blood–brain barrier is damaged, leukocyte infiltration, platelet aggregation and plasma exudation may jointly participate in the inflammatory response. After a week of implantation, microglia cells may clear a large amount of tissue debris through phagocytosis, and the tissue exudate absorption is also completed. Acute inflammatory response can be regarded as a process of repairing the tissue injury caused by the implantation process, and it would gradually subside after one or two weeks, which is a major cause of the malfunction and failure of the implanted electrodes.

After the acute response, the chronic inflammatory reaction phase begins, which features the presence of persistent inflammatory. In the chronic inflammatory reaction, the activated microglia may stick to the surface of the electrode, and the astrocytes are activated by the microglia, resulting in the formation of a glial scar around the electrode [34,38]. Microglia cell adhesion and aggregation on the electrode surface is the initial stage of chronic inflammatory response, and it takes place through the whole process of chronic inflammatory response. It has been found that the adhesion of microglia cells to the electrode surface was mediated by serum proteins [39]. In order to degrade and remove the foreign body (e.g., implanted electrodes), these activated microglia cells would secrete substantial decomposition enzymes and oxygen-free radicals, causing the death of the neurons around the electrode. In addition, the microglia may also secrete many cytokines, such as interleukin-1, interleukin-6 and tumor necrosis factor [40,41], which activate astrocytes. Astrocytes could also be activated by the source of the blood clotting enzyme, serum albumin and so on, which make the astrocytes proliferate and secrete an extracellular matrix, and inhibitory molecules, such as chondroitin sulfate proteoglycan [42]. Eventually, a dense layer of glial scar may form near the surface of the implanted electrode; this glial scar formation process generally takes about six weeks. As a result, the distance between the electrode and the target neuron increases, which further elevates the threshold value of the stimulation. Moreover, the formation of a glial scar around the electrode could inhibit the transmission of current and increase the impedance in the electrode–tissue interface. In order to achieve the optimal effect of the stimulation, the parameters for the electrical stimulation need to be adjusted, i.e., increasing the stimulating voltage, which further increases the energy consumption of the electrical stimulator, shortens the lifespan of the battery, and elevates the patient’s burden [43]. More importantly, the death of the neurons around the electrode caused by the inflammatory response may induce the failure of the electrode. For example, a study by McConnell et al. found that neuronal degeneration mediated by local chronic inflammation was also a major causation for the connection failure between the electrodes and neurons [44]. In this study, it was observed that the signal recording performance of the neural electrodes degenerated after the glial scar was stabilized (about six weeks). However, this degenerative process may continue even after six weeks. After carefully examining and comparing the electrode implanted for 16 weeks with the 8-week sample, McConnell et al. found that macrophages around the electrode increased, and the expression of the Tau protein phosphorylated at Thr-231 loci was also detected, indicating neuron degeneration, and the immunoreactivity change of PT231. In the brain tissue of senile dementia patients, the same phenomenon was observed. It was thus indicated that the macrophages were in a state of continuous activation due to the continuous existence of the electrode. It was also found that an inflammatory response mediated a state of neurodegeneration around the electrode, and the progressive death of the neurons may lead to the decline or even failure of the recording function of the electrode after the stabilization of the glial scar [45,46,47,48].

## 3. Metals and Their Derivatives

Metals such as platinum, gold and silver have been successfully applied in the fabrication of neural electrodes due to their excellent electrical conductivity, biostability and resistance to corrosion [49]. Moreover, they are also used for the fabrication of microelectrodes with smaller geometric sizes [50]. However, surface modifications are generally required for metallic electrodes in order to engineer the biomechanical, electrochemical, and foreign body response in the electrode–neural interface. Metallic materials and their derivatives such as gold and iridium oxide with nanostructures are widely used to modify the surface of microelectrodes, because they can easily form intact coatings on gold and metal electrodes in order to avoid parasitic effects like bimetallic corrosion or delamination [51,52]. On the other hand, metals and their derivatives show higher mechanical strength than other materials, which is important for the chronic implantation of neural electrodes [53].

### 3.1. Platinum Coatings

Metallic materials and their derivatives are usually employed as coatings on the surface of the conductive sites of neural microelectrodes. Recently, nanoparticles and porous structures composed of metallic materials and their derivatives were utilized as the coating layer, which can remarkably increase the geometric surface area of the metallic electrode and bring a significant improvement to the electrochemical properties of the microelectrodes [54,55,56,57,58].

**Figure 2:**
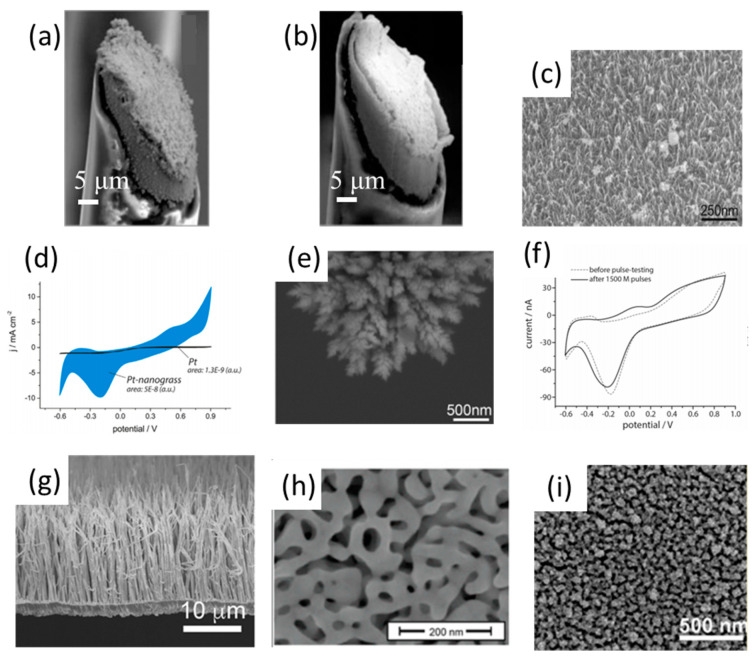
The SEM images of (**a**) the DC-plated platinum black, (**b**) the sonico-plated platinum black, (**c**) Pt-nanograss, (**e**) the NanoPt coating, (**g**) gold nanowires, (**h**) nanoporous gold, and (**i**) Au nanoparticles. (**d**) CV-measurements on 35-mm diameter electrode spots carrying the passive Pt-grass (blue squares) in comparison to sputtered Pt (black triangles). (**f**) The CV-characteristics show only marginal changes in response to the excessive pulsing (*n* = 5). (**a**,**b**) are reproduced with permission from [64], Copyright 2010, Frontiers. (**c**,**d**) are reproduced with permission from [71], Copyright 2015, Elsevier. (**e**,**f**) are reproduced with permission from [53], Copyright 2020, ACS Publications. (**g**) is reproduced with permission from [72], Copyright 2008, ACS Publications. (**h**) is reproduced with permission from [73], Copyright 2015, ACS Publications. (**i**) is reproduced with permission from [74], Copyright 2012, ACS Publications.

Platinum coatings formed by electrochemical deposition have gained much attention in neural electrodes. The rough platinum coatings with a high surface-to-volume ratio can effectively facilitate charge transfer and movement on neural electrodes. After the deposition of platinum coatings, the impendence of neural electrodes can be reduced at least four times [59,60,61,62], and the charge injection limitation of the neural electrode is increased by a factor of six [59,60], which is beneficial to obtain high-quality signal recording and ensure a more efficient and safer charge delivery for stimulation. However, while the enhancement in the electrochemical performance of the neural electrode is well documented for platinum coatings, other crucial parameters such as biocompatibility, durability, mechanical stability and stimulation performance remain to be explored. Few studies have reported that platinum black coatings (Figure 2a) had poor mechanical durability, which greatly hindered their applications for long-term implantation [63,64]. In order to enhance the stability of platinum coatings on neural electrodes, many methods have been reported, such as ultrasonic agitation [64,65,66], adhesion promoter additives [67] and substrate roughening [68]. The ultrasonic agitation method is widely used to improve the stability of platinum coatings (Figure 2b), i.e., the ultrasonically treated platinum coating only lost 2.5% of itself after the stability test, while there was about 80% lost for the untreated platinum coatings [65]. Moreover, the ultrasonic platinum coating showed excellent chronic stability, and there was no evidence of severe damage to the electrochemical property or failure after several months of implantation. Other than the ultrasonic agitation method, Yu et al. presented a method to electroplate highly adhesive platinum black onto a microelectrode by using adhesion-promoter additives, which showed even better durability than those prepared by the ultrasonic agitation method [67]. According to their method, a fuzzy gold coating was first coated onto the electrode as the intermediate layer to enhance the adhesion of platinum coating on the microelectrode sites. As a comparison, both the ultrasonic-plated platinum black and the fuzzy gold interlayered platinum black coatings showed decreased the effective surface area and significantly increased the impedance. The fuzzy gold interlayered platinum black coatings maintained a larger effective surface area (77%) than the ultrasonic plated platinum black. Although the platinum black coating formed by using traditional electroplating could enhance the neural electrode interface, the cytotoxicity of the platinum black is a serious concern. Few studies reported that the DNA synthesis of rat oligodendrocytes in vitro was inhibited when exposed to the platinum black extract [69]. The release of toxic lead, an ingredient of the conventional electroplating electrolyte, may be responsible for this issue [70]. Therefore, many new methods have been developed for the formation of the nanostructured platinum coating on the neural electrode interface.

The three-dimensional nanostructured platinum coating can be fabricated by chemical or electrochemical depositions, which do not contain cytotoxic components like lead. There were no toxic effects of the extract products of the coatings fabricated by these two methods on cells during in vitro experiments [71]. Chemical deposition can endow the platinum coatings with nanowire-like structures and a higher effective surface area, while electrochemical deposition could form clear specific patterns of coatings efficiently. Boehler et al. introduced a chemical deposition method for the formation of nanostructure platinum grass to enhance the neural electrode interface [71]. Because of the high effective surface area of the platinum nanograss (Figure 2c), the electrochemical property of the coating was significantly improved (Figure 2d), with a significant decrease of the impedance of almost two orders of magnitude, and a dramatic increase in the charge delivery capacity. The nanograss coating was non-toxic, as confirmed by an elution testing spanning over the different concentration levels. Although the effective surface area can be increased, coatings formed by a chemical reduction process were highly time-dependent and hard to control due to the limitations of the chemically reducing agent, which prevented the fabrication of application-specific coating morphologies and substantially limited the reproducibility and the mass production of the coated electrodes. A new electrochemical deposition method without the chemically reducing agent for the formation of a nanostructure platinum (nanoPt) coating (Figure 2e) may be possible in order to substantially improve the process control that is vital for large-scale fabrication [53]. The morphology of the electrochemically deposited nanoPt coatings could be selectively modified by dynamically varying the deposition voltage during the electrochemical reduction process. The nanoPt coating can maintain its stability over more than 1 billion stimulation pulses under a charge density of 1.5 mC cm^−2^ (Figure 2f).

### 3.2. Gold Coatings

In addition to platinum, gold (Au) with micro-/nano-structures is also used as a coating for neural microelectrodes. Gold coatings could be fabricated by many methods, such as chemical reduction [75], evaporation [76] and sputtering [77]. In order to enhance the electrochemical property of the neural microelectrodes, templates with nanoscale structures were used to increase the effective surface of gold coatings [72,75,78]. Nakanishi et al. used the anodic aluminum oxide template to fabricate gold nanowires through an electrodeposition technique [72]. The vertically aligned structure of the nanowires was observed by SEM (Figure 2g). Tybrandt et al. employed titanium dioxide nanowires as a starting material to fabricate gold-coated titanium dioxide nanowire (Au-TiO_2_ NWs) coatings for neural microelectrodes. The electrode coated with Au-TiO_2_ NWs had a high areal capacitance, with 2.7 mC cm^−2^, and it also showed stable neural recording after implantation for 3 months [75]. Dealloying was used to form a gold coating with a higher porosity and interconnectivity [73,79,80,81]. In a typical process, an Ag-Au alloy coating was first co-deposited on the electrode; subsequently, Ag was selectively dissolved to form the nanoporous gold coating (Figure 2h). The impedance of the nanoporous gold coated electrode was decreased by more than 25 times, and the biocompatibility of these coatings was also good [73]. The direct deposition of gold nanoparticles and substrate roughening were also utilized to improve the effective surface area of the gold coating [74,76]. The layer-by-layer (LBL) assembly of the Au nanoparticles (NP) (Figure 2i) achieved superior improvements on the electrical conductivity and charge transfer properties of the electrode as compared to CNT coatings. The good adherence, viability and differentiation of cultured neurons indicated that the LBL Au coating was also biocompatible [74]. Moreover, in order to achieve the high-density adhesion of neural cells to the neural microelectrode conductive sites, Mescola et al. proposed a two-step functionalization method to endow the neural cells with selective-adhesion ability to the gold surface, which provided an effective way to construct a stable electrode–nerve interface between neural cells and gold microelectrodes [82].

### 3.3. Iridium Oxide

**Figure 3:**
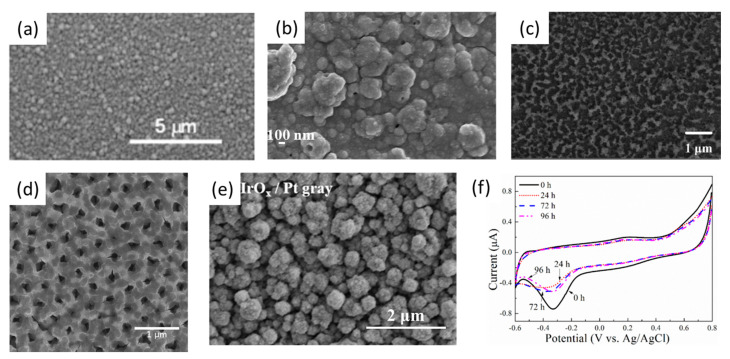
SEM image of (**a**) SIROF, (**b**) EIROF, (**c**) AIROF, (**d**) nanoporous AIROFs, and (**e**) IrO_x_/Pt gray. (**f**) Cyclic voltammograms of an IrO_x_/Pt gray-coated microelectrode before and after 24 h, 72 h and 96 h of continuous stimulations. (**a**) is reproduced with permission from [85], Copyright 2009, Wiley Online Library. (**b**) is reproduced with permission from [97], Copyright 2009, Elsevier. (**c**) is reproduced with permission from [107], Copyright 2014, Elsevier. (**d**) is reproduced with permission from [105], Copyright 2010, Elsevier. (**e**,**f**) are reproduced with permission from [106], Copyright 2017, Elsevier.

Due to platinum and gold’s faradaic nature, their limitations of charge delivery capacity remain a great challenge, especially for the electrodes with small geometric surface areas. With weak or no faradaic reaction charge transduction, the contribution of double-layer capacitive coupling in charge delivery is often limited [83]. Thus, materials with higher electroactivity and ability to support reversible faradaic reactions are needed to promote the charge transfer in the electrode–tissue interface. Iridium oxide (IrO_x_) is a good candidate material for neural electrode coatings due to its low impedance, high charge storage capacity and charge injection capacity [55,84,85,86,87]. IrO_x_ also shows good biocompatibility and high corrosion resistance [56,88]. The neural microelectrode coated with IrO_x_ displayed an excellent stimulating function for individual neurons and recorded multiple single-unit spike activity with good signal-to-noise ratios [89,90]. The structure and properties of IrO_x_ are different based on the fabrication methods [91]. There are many kinds of iridium oxide, including sputtered iridium oxide film (SIROF) (Figure 3a) [85,87,91,92,93,94], electrodeposited iridium oxide film (EIROF) (Figure 3b) [84,95,96,97,98], activated iridium oxide film (AIROF) (Figure 3c) [86,99,100,101,102], atomic-layer-deposited iridium oxide film [103], and physical-vapor-deposited iridium oxide film [104]. A porous iridium oxide layer could be observed for AIROF and EIROF, while SIROF showed a dendritic surface structure. AIROF and EIROF showed a higher charge storage capacity and lower impedance than SIROF for similar thicknesses, because AIROF and EIROF had a more open and porous structure, but SIROF exhibited better performance in terms of electrochemical and mechanical stabilities than AIROF. SIROF was more durable than AIROF under continuous high-charge-density stimulations, and SIROF was also found to keep its electrochemical property in ambient conditions which lasted for a month. In order to enhance the electrochemical performance of IrO_x_ coatings, electrochemical activation was employed to endow the IrO_x_ coatings with higher porosity and a larger effective surface area.

However, it is contradictory that the electrochemically activated iridium oxide coating obtained better electrochemical performance at the expense of its mechanical properties, which are important for the chronic implantation of a neural microelectrode. Other metals and metallic oxides were also used as templates or additives to improve the functions of the IrO_x_ coating [78]. David et al. utilized a nanoporous anodized aluminum oxide (AAO) template to produce AIROF [105], which possessed a high charge storage capacity of more than 300 mC cm^−2^ and good mechanical stability (Figure 3d). The impedance of the gold nanowires modified with an EIROF coating was found to decrease by about three orders of magnitude, and the modified neural electrode had a good performance in recording neural spikes in vivo [78]. Zeng et al. combined iridium oxide with platinum gray to fabricate EIPOF/Pt gray composite coatings [106]. Due to the large surface area of the nanocone-shaped Pt gray (Figure 3e), iridium oxide could firmly adhere to the microelectrode, which showed superior mechanical and electrochemical stability (Figure 3f). An ultrasound bath was employed to test the mechanical stability of the EIPOF/Pt gray coating. The impedance of the EIPOF/Pt gray coating was stable at 3.5 kΩ after ultrasonic treatment (50 W) for 1 h, which was about 15 times lower than bare Pt.

## 4. Carbon Materials

Although nanostructured metals and their derivatives could be used to construct the coating layers of neural electrodes, which can increase the effective surface area and improve the electrochemical properties of the neural interface, the stability issues of metallic coatings in vivo have raised serious concerns. For example, platinum black with a porous, low-impedance structure is mechanically fragile and degradable in the physiological environment [108]. An activated IrO_x_ coating with excellent charge transfer properties was used for neural stimulation, but the surface of it was found to be chemically unstable [109]. Carbon materials, such as carbon nanotubes (CNT) and graphene, are recognized as promising candidates to compromise the disadvantages of the metallic coatings, due to their lower toxicity, larger surface area, excellent electrical properties, and biocompatibility [110,111,112,113,114]. CNTs and graphene have been confirmed to be promising coatings for neural electrodes by numerous studies, as described in the following sections.

### 4.1. Carbon Nanotubes

Carbon nanotubes are composed of rolled-up graphene sheets, which can be classified into two types based on their wall structure, i.e., single-wall carbon nanotubes (SWCNTs) and multi-wall carbon nanotubes (MWCNTs) [115]. Due to their large surface area, and good electrical and physical properties, such as high conductivity and a high aspect ratio [116], carbon nanotubes are used as superior coating materials for neural microelectrodes, which have shown low impedance, high charge transfer mobility, chemical stability, and biocompatibility. The reported impedance values of neural electrodes coated with CNTs are lowered by 10 to 60 times compared to those of the bare electrode [52,112,117,118]. In addition, due to the high surface-to-volume ratio of CNTs, the charge storage capacity (CSC) of the CNT-coated neural electrode can be increased by about 3–140 times, which is higher than PEDOT and IrO_x_ with the same thickness [119,120]. Because of the excellent mechanical strength and ductility of CNTs, CNT films could strongly adhere to the surfaces of microelectrodes [121,122]. Moreover, CNT-coated electrodes showed good stability in mechanical and electrochemical tests. For example, after hundreds of cyclic voltammetry (CV) tests, the CNT-coated electrode exhibited a minimal loss of electrochemical properties compared to the conducting polymer coatings, and kept its structure intact when the PEDOT coatings broke and formed cracks [123].

**Figure 4:**
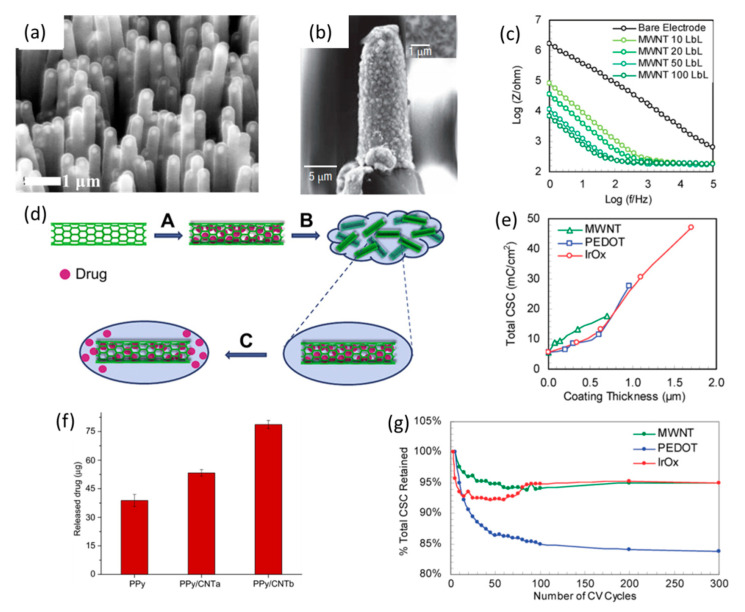
SEM images of (**a**) an as-grown carbon nanofiber array and (**b**) CNT coating by covalent attachment. (**a**) is reproduced with permission from [135], Copyright 2006, Wiley Online Library. (**b**) is reproduced with permission from [119], Copyright 2008, nature.com. (**c**) EIS of CNT coatings with different thicknesses. (**d**) Schematic of the drug loading and release process of CNT nano-reservoirs. (**e**) The total CSC of CNTs, PEDOT and IrO_x_ coatings. (**f**) Dex released from different PPy films under electric stimulation for 20 h. The electric stimulation applied was a 50% duty cycle of square wave, 2 V for 5 s followed by 0 V for 5 s. The data are shown ± the standard deviation (*n* = 3 for each case). (**g**) The total charge storage capacities (CSC) of the CNTS, PEDOT and IrO_x_ coated electrodes were recorded while being subjected to 300 cyclic voltammetry scanning cycles. (**c**,**e**,**g**) are reproduced with permission from [117], Copyright 2009, ACS. (**d**,**f**) are reproduced with permission from [145], Copyright 2011, Elsevier.

CNT coatings can be deposited on the conductive sites of the neural electrodes by a variety of methods, including chemical vapor deposition (CVD) [124,125,126,127,128], electrochemical deposition (ED) [129], microwave plasma [130], layer-by-layer assembly (LBL) [131,132,133], solvent evaporation [134], and covalent attachment [119]. The physical, chemical and bio-properties of the CNT coatings can be determined by the fabrication methods. CVD is one of the most widely used methods to produce CNTs. CNTs synthesized by CVD can be grown to highly porous mats or highly ordered and vertically aligned pillar bundles by applying different lithographic patterns of catalyst [128]. The highly porous CNTs with a fluffy mat structure have a larger surface area for charge transfer and the preferential adhesion of neural cells via dendrite entanglement to the carbon nanotubes, which can significantly improve the signal recording of the neural electrode [118]. The CNT pillar coating generated by CVD also exhibited excellent mechanical stability and electrochemical properties. Nguyen-Vu et al. fabricated vertically aligned carbon nanofiber arrays (Figure 4a) to improve the performance of neural microelectrodes through the construction of a three-dimension interface between the microelectrodes and local neural tissues [135]. The as-prepared CNT array was a unique, highly porous 3D material with a large surface area. When bent by the cell bodies, the breakage and delamination of the CNTs from the electrode sites during the in vitro tests were not observed, indicating the superior flexibility and strength of the highly order and vertically aligned carbon nanotube pillar bundle coatings. The structure of the aligned CNTs was similar to the highly oriented pyrolytic graphite, and thus the aligned CNTs were shown to have faster electron transfer kinetics than the randomly oriented CNTs [136]. However, the randomly oriented CNTs may exhibit a better electrochemical property than aligned CNTs because of their enhanced porosity. The aligned CNT-coated electrodes had extremely low impedances and provided a roughened surface that produced excellent cell–electrode coupling [137]. However, the deposition of CNTs by CVD requires a high temperature (400–900 ℃), which is only applicable to thermally stable electrode and substrate materials.

Compared to CVD, electrochemical deposition has several advantages because the coating process is simple and could be completed in ambient conditions and a mild solution. The structures and properties of CNT coatings obtained by electrochemical deposition can be controlled by parameters such as the CNT concentrations, deposition charges and choice of electrolytes. The CNT coatings fabricated by electrochemical deposition are porous but fragile, which would limit their electrochemical performance during long-term implantation [138]. Moreover, this problem also occurs in CNT coatings made by simple solvent evaporation due to the planar morphology of the coating [134]. In order to enhance the stability of the CNT coating on a neural electrode, covalent attachment is a preferable choice. The coherent and stable CNT coatings can adhere to the electrode sites through covalent bonds. The study by Keefer et al. showed that the parylene insulation was peeled back from the electrode and rolled up to the shaft by the mechanical stress while the covalently attached CNTs remained intact during the implantation (Figure 4b) [119]. Implantable neural microelectrodes require superior mechanical properties in order to withstand chronic stresses and harsh physiological conditions. Furthermore, the layer-by-layer (LBL) assembly technique is also applied to fabricate multilayered composites of CNTs and polyelectrolytes, which is attractive for the construction of a robust neural interface. The surface morphology of the CNT coatings obtained by LBL is composed of uniformly dispersed CNT bundles with nanoscale roughness, which can significantly increase the surface area, improve the charge transfer efficiency, and provide superior structural stability. The thickness of the LBL CNT coatings can be controlled with precision by the number of layers of CNTs and polyelectrolytes, respectively. Jan et al. reported that the LBL MWCNT coating showed better performance than PEDOT and IrO_x_ films in terms of impedance, charge storage capacity and charge transfer efficiency. The uniform coating made by LBL assembly could transfer both ions and electrons, and no signs of failure of the charge storage capacity was detected after 300 CV cycles, showing that the coating was highly stable under the in vitro electrochemical conditions (Figure 4c,e,g) [117].

Although the CNT coatings have shown significant capabilities in improving the functions of neural electrodes, the toxicity issues are essential. Few studies reported that the aggregation of CNTs in the surrounding tissues may cause severe tissue damage [139,140]. CNTs modified with natural or synthetic biocompatible polymers could overcome such problems. Cho et al. prepared a series of electrically conductive CNT/collagen composites to study their cellular responses when PC12 cells were grown on this composite film with or without electrical stimulation [141]. The results indicated that as the content of collagen increased, PC12 cells showed enhanced attachment on the CNT/collagen composite due to the ability of collagen to improve the adhesion and viability of the nerve cells. The CNTs in the hyaluronic acid (HA) nanofiber composites proposed by Steel et al. were proven to enhance and accelerate the regenerative behaviors of the neurons under safe and effective electrical stimulations [142]. The CNT coatings encapsulated by biocompatible boron-doped diamond were shown to promote the growth of cells and reduce the unfavorable release of CNTs into the surrounding tissues [143]. The surface of the CNT coatings functionalized by amino groups were also proven to support the adhesion and growth of neuronal cells in vitro [144]. Moreover, CNT coatings functionalized with other molecules, such as nucleic acids, drugs and proteins, have been used as controllable drug release systems. For example, Cui’s group used MWCNTs as nano-reservoirs for drug delivery (Figure 4d) [145]. To speak further, the inner cavity of MWCNTs can be filled with a dexamethasone solution and encapsulated in polypyrrole (PPy) thin films. Compared to the conventional PPy thin film, the MWCNT coating displayed a higher drug loading capacity and a more linear and continuous release profile under electrical stimulations (Figure 4f) [145].

### 4.2. Graphene

**Figure 5:**
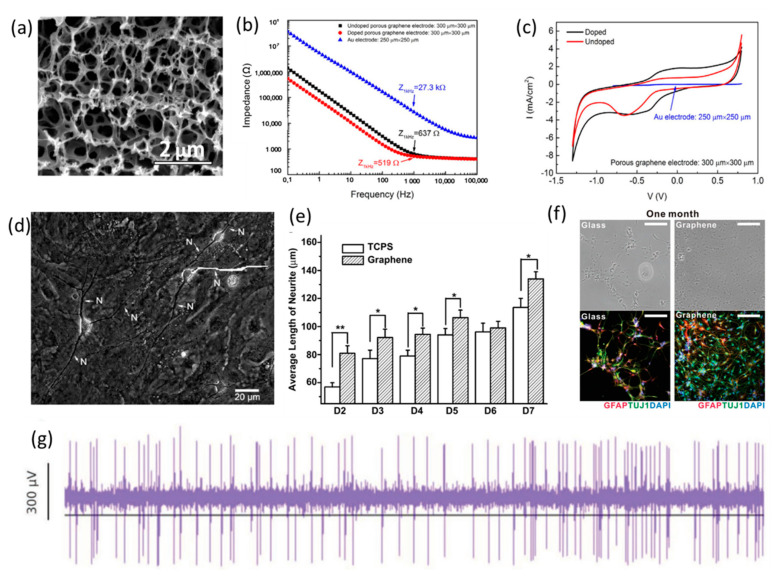
(**a**) SEM image of the 3D porous graphene. (**b**) EIS of a comparison of doped and undoped porous graphene and Au electrodes. (**c**) CV-characterization of porous graphene and Au electrodes. (**a**–**c**) are reproduced with permission from [158], Copyright 2016, Springer Nature. (**d**) Phase-contrast micrograph of typical neurons showing a trace along the extension of neurite (N) used for length calculation, (**e**) average number of neurites per neuron on TCPS and graphene during the developing period (D2–D7). (**d**,**e**) are reproduced with permission from [148], Copyright 2011, Elsevier. (**f**) Bright-field (top row) and fluorescence (bottom row) images of hNSCs differentiated on glass (left) and graphene (right) after one month of differentiation. The differentiated hNSCs were immune-stained with GFAP (red) for astroglial cells, TUJ1 (green) for neural cells, and DAPI (blue) for nuclei. Note that more hNSCs were adhered to graphene than to glass. All scale bars represent 200 μm. (**f**) is reproduced with permission from [169], Copyright 2011, Wiley. (**g**) Representative voltage time trace of graphene-coated NeuroNexus probe. (**g**) is reproduced with permission from [168], Copyright 2019, Wiley.

Graphene has recently become an attractive candidate for neural interfacing owing to its superior chemical and physical properties, including its high conductivity, good chemical stability, flexibility, transparency and biocompatibility. In recent years, the studies of neuron networks cultured on graphene-based neural electrode coatings have shown that graphene was compatible to neural cells [146,147,148]. The fabrication methods of graphene coatings on neural electrodes include graphite exfoliation [149,150,151], electrochemical exfoliation [152,153,154] and CVD growth [155,156,157]. Due to the unique 2D structural features associated with its large specific surface area and superior electrical conductivity, the graphene coating can remarkably improve the electrochemical properties of neural electrodes. However, the charge storage capacity of graphene was limited due to its flat surface. Increasing the surface roughness of the graphene coating is an effective approach to improve the efficiency of the micro-stimulations delivered by the neural microelectrodes. A 3D porous graphene coating (Figure 5a) was produced by Lu et al. though the direct laser pyrolysis method [158]. The electrode-coated porous graphene exhibited a high charge injection capacity (CIC) of 3 mC cm^−2^ and an impedance lower than a gold electrode with similar sizes (Figure 5b,c). The in vivo test results showed that high density porous graphene electrode arrays could deliver electrical micro-stimulations to map cortical areas with both high resolution and high precision (Figure 5g). Due to its transparency, neural electrodes based on a graphene layer have been applied for simultaneous electrophysiology and neuroimaging [159]. Graphene-coated copper microwires that are highly compatible with magnetic resonance imaging (MRI) can be applied in the recording of brain activities and clinical diagnoses [21].

The in vitro studies showed that graphene could support the growth of neural cells and regenerate the targeted, damaged neurons, rather than inducing the proliferation of reactive astrocytes which could reduce the lifetime of the implanted electrodes [160,161,162,163,164]. Bendali et al. cultured purified adult (8-week old) retinal ganglion cells (RGC) on bare graphene in order to study the growth process of neurons directly in contact with graphene. The results indicated that neurons with outgrown neurites could easily survive on the non-peptide-coated graphene, showing the high cytocompatibility of graphene. Additionally, the graphene substrates were reported to promote the neurite sprouting and outgrowth of mouse hippocampal cells after 7 days of culture (Figure 5d,e) by Li et al. [148]. Furthermore, few studies focused on the tissue response to graphene coatings in vivo and the correlative effects on the detection efficiency and time reliability of neural electrodes [165,166,167]. The work from Bourrier et al. indicated that the proliferations of astrocytes and microglia were significantly reduced around the monolayer graphene-coated probes after 5 weeks of implantation, suggesting that graphene was associated with the reduction of the tissue response due to its flexibility and function as a diffusion barrier [168]. Moreover, graphene was also able to enhance the neuronal differentiation of human neural stem cells (hNSCs). The hNSCs exhibited excellent adhesion on graphene during the long-term differentiation process, and the differentiation of hNSCs may progress toward neurons rather than glial cells on graphene (Figure 5f) [169].

## 5. Conducting Polymers

Due to their versatile chemical structures and tunable surface functionalities, polymers have been utilized as advanced materials for biomedical engineering. Among the synthetic polymers, conducting polymers (CPs) are electroactive and nontoxic, and have received considerable attention as promising coatings for neural interfacing [170,171].

CPs originated from the discovery of polyacetylene in the late 1970s [172]. Subsequently, the discovery and development of CPs won the Nobel Prize in chemistry in 2000 [173,174,175]. The alternate single- and double-bond conjugating backbones form the molecular structure of CPs, facilitating electronic conductivity after doping with electron donor or acceptor dopants [176]. Because of their low elastic modulus, good biocompatibility and excellent conductive properties, CPs have been employed in a wide range of energy storage and biomedical applications, such as supercapacitors [177,178], biosensors [179,180], tissue engineering [181,182] and drug delivery systems [183,184]. Recently, many studies have focused on the applications of CPs in neural interfacing [185,186,187,188,189,190].

CPs—such as polypyrrole (PPy), polyaniline (PANI), poly(3,4-ethylenedioxy thiophene) (PEDOT) and their derivatives—have been widely used in biomedical applications. Among CPs, PPy and PEDOT have received more interest in neural interfaces due to their biocompatibility, high electron and ion conductivity, and ease of coating. The synthesis of CPs can be accomplished by solution precipitation and electrochemical polymerization [17,191,192,193]. Generally speaking, the yield of CPs synthesized by solution precipitation is higher than that of electrochemical polymerization, but it is hard to directly apply a homogeneous CP coating onto a metallic substrate through solution precipitation [191]. The electrochemical deposition of CPs is widely used to fabricate well-defined CP thin films on the electrode surface, as the thickness, surface structure and electrical conductivity of the CP film can be readily controlled by adjusting the electrochemical parameters [17,191,192].

**Figure 6:**
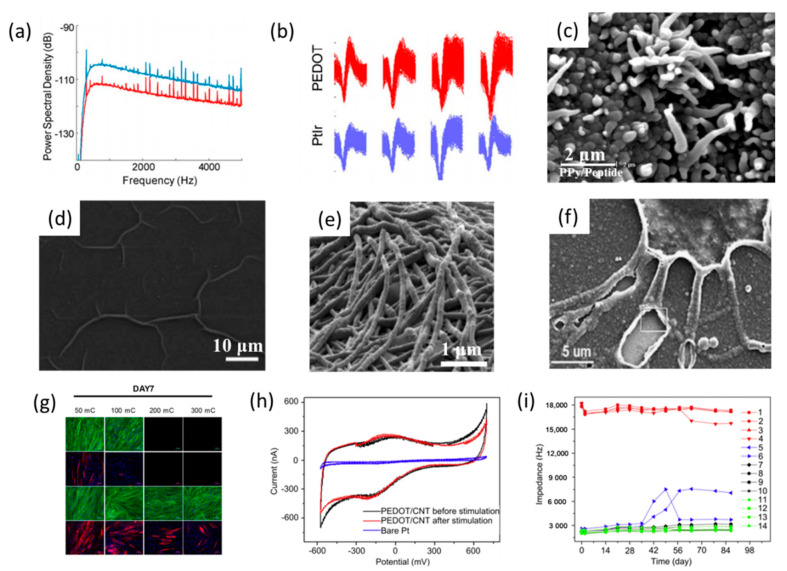
(**a**) Average power spectral density measured on the PEDOT and PtIr electrodes during surgery under high isoflurane. (**b**) 100 overlaid action potential waveforms (0.8 ms for each) recorded on different electrodes of the same array. (**a**,**b**) are reproduced with permission from [192], Copyright 2011, IEEE. The SEM image of the (**c**) PPy/DCDPGYIGSR, (**d**) PPy-NGF, (**e**) PEDOT nanotubes, and (**f**) cell-templated PEDOT coating. (**c**) is reproduced with permission from [200], Copyright 2003, Elsevier. (**d**) is reproduced with permission from [202], Copyright 2007, Wiley. (**e**) is reproduced with permission from [206], Copyright 2008, Elsevier. (**f**) is reproduced with permission from [187], Copyright 2007, Elsevier. (**g**) Immunofluorescence images of C2C12 cells cultured on PPy and PDA/PPy samples for 7 days. The cells were stained for F-actin (green), MHC (red) and nuclei (blue). Scale bars = 200 μm. (**g**) is reproduced with permission from [203], Copyright 2018, ACS. (**h**) Cyclic voltammograms of a bare Pt electrode and PEDOT/CNT coated electrodes before and after long-term stimulation. (**i**) Monitored electrode impedance changes at 1 K Hz over time during three months of soaking in PBS. Plots 1–4 in red are bare Pt electrodes; plots 5–6 in blue are ultrathin PEDOT/CNT-coated (deposition charge less than 5 mC) Pt electrodes during stimulation; plots 7–10 in black and plots 11–14 in green are normal PEDOT/CNT-coated (deposition charge more than 10 mC) Pt electrodes with (7–10) and without (11–14) stimulations. (**h**,**i**) are reproduced with permission from [207], Copyright 2011, Elsevier.

Electrodes coated with CPs have shown superior electrochemical properties, such as lower impedance, higher CSC, a higher charge injection limit (CIL), and better biocompatibility than uncoated electrodes. Cui et al. modified the conductive sites of neural electrodes by the electrochemical deposition of PPy doped with polystyrene sulfonate (PSS) [17]. The results indicated that the impedance of the PPy/PSS-coated electrode was 30 times lower than a bare gold electrode at the biologically related frequency of l kHz, which can be attributed to the enhanced electrochemically active surface area created by the PPy coating. A study by Venkatraman et al. indicated that a PEDOT-coated electrode showed a CIL 15-times higher than the same IrO_x_ coating at zero voltage bias [192]. The CP-coated electrode also exhibited remarkable performance in neural signal recording and stimulation in vivo. Martin’s group found that the SNR (5.1 ± 1.2) of the signals recorded on PEDOT-coated neural probes was significantly higher than the SNR (4.3 ± 1.0) of the signals recorded on uncoated electrode sites after 15 days of implantation (Figure 6a,b) [192]. Furthermore, other in vivo studies also showed similar results, i.e., that the CP-coated electrodes can significantly increase the SNR and the population of neural cells recorded [193].

For electrochemically polymerized CP films, the types of dopants may have a significant impact on their structural, chemical and physical properties. Many dopants have been used in the literature, such as sodium chloride (NaCl) [32], lithium perchlorate (LiClO_4_) [194], sodium benzenesulfonate (BS) [194], sodium p-toluenesulfonate (pTS) [195], tetrabutylammonium perchlorate (TBAP) [196], sodium dodecylbenzene sulfonate (DBS) [197], heparin [198] and poly(sodium 4-styrenesulfonate) (PSS) [32]. CPs polymerized with small dopants such as LiClO_4_ showed high structural regularity, a more compact internal structure, and higher conductivity and flexibility compared to those doped with larger dopants such as PSS and heparin [32,194,198]. The use of small-sized dopants, a high deposition charge density, and a high dopant concentration in the electrochemical polymerization solution was able to provide a rougher coating surface morphology [194]. However, the work from Pool-Warren and co-workers showed that dopants with larger sizes may generate softer and less-adherent CP films [199]. The uniformity of the coating on the electrode sites also varies with the size of the dopants. PSS-doped PEDOT showed aggregation on the edge of the electrode sites, while ClO_4_^-^ and pTS-doped CPs showed more even coatings [194,195]. In order to improve the biocompatibility of CPs and reduce the tissue responses during implantation, bioactive molecules can be used as dopants and incorporated in the CP coating during electrochemical polymerization. Biologically active dopants—such as laminin peptide sequences, hyaluronic acid, or silk-like polymers with fibronectin fragments (SLPF)—can potentially enhance the biocompatibility and the cell adhesion on the surface of neural electrodes [200,201,202]. Cui et al. co-deposited PPy and synthetic-peptide DCDPGYIGSR on the electrode surface by using electrochemical polymerization (Figure 6c) [200]. The immunocytochemical studies indicated that the density of neurofilaments found by positive staining on the coated electrode was significantly higher than that on the uncoated electrode, indicating that the PPy/DCDPGYIGSR coating established strong interactions with the neuronal structure in vivo. The PPy film can significantly enhance the neurite outgrowth of PC12 cells by incorporating brain-derived nerve growth factor (BDNF) and nerve growth factor (NGF) [201]. Gomez and co-workers immobilized NGF on the surface of PPy using an intermediate linker, i.e., a layer of polyallylamine conjugated to an arylazido group (Figure 6d) [202]. Upon the application of electrical and chemical stimuli, the neurite lengths of PC12 cells cultured on the PPy-NGF film were much longer than the ones on the PPy film. Kim et al. reported the modification of the neural electrodes by the electrochemical copolymerization of polydopamine (PDA) and PPy to achieve enhanced biological performance [203]. PDA/PPy films were shown to have higher cell attachment and faster cell growth than the PPy films after 7 days of culturing. After the application of electrical stimulations, PC12 cells cultured on the PDA/PPy showed a significant promotion in neurite formation compared to the PPy films (Figure 6g). In addition to proteins and NGF, anti-inflammatory drugs like dexamethasone (DEX) can also be incorporated and released from CP coatings to reduce the tissue responses with improved electrode–tissue interactions [204].

The structural features of the CP coatings, such as their surface morphology and pore structure, play important roles in determining their electrical, mechanical and biological properties. A larger effective surface area is beneficial for the charge transfer between the electrode and electrolyte interface. Much research on the structural design and surface functionalization of the CP coatings were implemented to improve their performance. Martin’s group employed monodispersed polystyrene latex spheres as templates to synthesize microporous PEDOT and PPy coatings on neural electrodes [205]. The corresponding electrochemical tests indicated that the impedance value of porous and rough CP-coated electrodes was significantly lower than the bare gold electrodes over the whole range of frequencies, which coincided with an increase in the effective surface area because of the rough surface morphology. A nanotube-like CP coating was produced using electrospun nanofiber as a template. Firstly, poly(L-lactide) (PLLA) was deposited on the neural electrodes by electrospinning to form a nanofiber-templating layer. Then CP was electrochemically deposited on the PLLA nanofiber-coated neural electrodes. Afterwards, the PLLA nanofibers were removed using dichloromethane, leaving the CP nanotube-coated neural-coated electrode (Figure 6e) [206]. The impedance of the neural electrode was sharply decreased in the presence of the CP nanotubes. During the in vitro and in vivo recording tests, CP nanotube-coated neural electrodes showed more effective recording sites than the uncoated electrodes. In order to increase the integration in the electrode–tissue interface, a biomimetic, neuron-templated CP coating was reported by Richardson-Burns and coworkers [186]. They electrochemically deposited PEDOT around living cells, and the cells were removed from the PEDOT coating using enzymatic and mechanical disruption to form a cell-templated PEDOT film (Figure 6f). They speculated that the cell-templated surface with biomimetic topologies would be more biocompatible and cell-attractive.

Although CP coatings can reduce the impedance and increase the charge storage capacity of neural electrodes, the relatively weak electrochemical and mechanical stability of CP coatings remain a great challenge for chronic recording and stimulations. In order to improve the mechanical durability of the CP coatings, CNTs and graphene oxide have been used as the reinforcing materials in CP films. Due to the mechanically robust and highly conductive CNTs and graphene, the carbon–CP composite coatings showed both strong mechanical stability and high conductivity (Figure 6h,i) [207,208]. The adhesion of CP to a neural electrode can also be enhanced by roughening the surface of neural electrodes in a pretreatment step [209]. Recently, PDA has also been utilized to improve the interaction between CP coatings and the neural electrode [210].

## 6. Hydrogels

**Figure 7:**
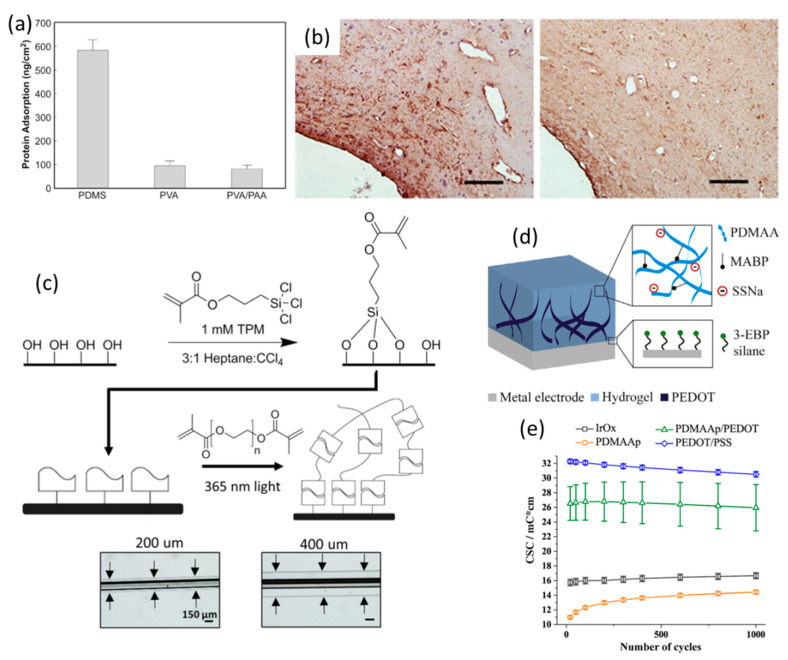
(**a**) Non-specific adsorption of protein (fibrinogen) on PDMS without a coating, with PVA coatings, and with PVA/PAA IPN (PAA/PVA = 10 mol%) film coatings (*n* = 3). (**b**) GFAP immunostaining of uncoated (left) and PVA/PAA IPN (PAA/PVA = 10 mol%) film-coated (right) PDMS implants after six weeks post-implantation (bar = 50 mm). (**a**,**b**) are reproduced with permission from [213], Copyright 2009, Elsevier. (**c**) Soft PEG hydrogel coatings were formed on borosilicate glass capillaries through a multistep process. (**c**) is reproduced with permission from [48], Copyright 2017, Springer-Nature. (**d**) Schematic depiction of the CPH composition. The hydrogel component PDMAAp consists of the PDMAA backbone, the MABP crosslinker and the SSNa units, which serve as counterions during the deposition of PEDOT. The hydrogel is covalently attached to the surface with 3-EBP silane. (**e**) The mean CSC of the different coating materials is shown vs. repetitive cycling (*n* = 1000 cycles) in PBS. (**d**,**e**) are reproduced with permission from [217], Copyright 2017, Elsevier.

Although the electrochemical properties of neural electrodes coated with nanostructured metallic, carbon-based and conducting polymeric materials have been significantly improved, the issue of the mechanical mismatch between electrodes and tissues, which may cause a series of inflammatory responses, still remains. Hydrogels, which have similar mechanical properties to biological tissue, high water contents, and good biocompatibility, have been considered as a promising coating candidate for neural interfacing. Hydrogels with optimal mechanical properties can serve as a buffer layer between the hard electrodes and soft tissues in order to reduce the adverse tissue responses induced by brain micromotion. Alginate [208], poly(ethylene glycol) (PEG) [48,211] and poly(vinyl alcohol) (PVA) [212,213] hydrogels have been employed to reduce the degree of glial scarring and neural cell loss. Lu et al. synthesized poly(vinyl alcohol)/ poly(acrylic acid) interpenetrating polymer network (PVA/PAA IPNs) hydrogel coatings and investigated their feasibility for implantable neural electrodes [213]. PVA was chosen due to its excellent mechanical strength, good film formation property and stability in physiological conditions. The results of the protein adsorption tests indicated that the fibrinogen adsorption on the PVA/PAA IPNs coating was approximately seven times lower than the uncoated surface, as a result of the protein resistive PAA (Figure 7a). The non-specific adsorption of protein layers is considered harmful to the performance of neural electrodes. The number of astrocytes around the PVA/PAA IPN-coated implant was significantly lower than that of the control group after 6 weeks of implantation (Figure 7b). Lu et al. also investigated the effects of polyethylene glycol-containing polyurethane hydrogel coatings and polyurethane -poly(vinyl alcohol) hydrogel coatings in improving the biocompatibility of neural electrodes [211]. Furthermore, the incorporation of neurotrophins, neural adhesion molecules and anti-inflammatory drugs into hydrogel coatings has been proven to reduce the degree of astrogliosis and the loss of neuronal bodies around the neural implants.

However, due to the low electrical conductivities of pure hydrogel-based coatings, the electrochemical performance of the non-conducting hydrogel-coated electrodes was not significantly enhanced. For example, PVA-based and PEG-based hydrogel coatings may bring higher impendence values to the underlying neural electrodes. New strategies to enhance the electrochemical properties without compromising the advanced biomechanical features of hydrogel coatings can provide reliable solutions to further strengthen the tissue–electrode interfaces. Due to their nano- and micro-porous 3D polymeric network structures, the apparent properties of hydrogels, e.g., electrical properties, can be readily adjusted by adding different functional materials. Many electrically conducting materials—including CNTs, graphene, and conducting polymers—have been added into the hydrogel matrices to improve the electrical conductivity. Among the various conductive hydrogel coatings, conducting polymer-based hydrogel coatings have been widely used in neural interfacing, owing to their good electrochemical properties, biocompatibility, and ease of processing and preparation. Conducting polymers can be crosslinked with non-conducting hydrogel templates to form interpenetrating hydrogel networks (IPNs). With the addition of conducting polymers, the electrochemical properties of the hydrogel coating can be significant improved. Different types of hydrogels (such as the alginate hydrogel (HG), PEG hydrogel and polyacrylamide hydrogel) and conducting polymers (such as PEDOT, PPy and PANI) have been employed to form IPN hydrogel coatings for neural electrodes. Works by Kim et al. indicated that as PEDOT was deposited on the conductive sites of the neural electrode, the number of clearly detectable units and the values of SNR for the HG-coated electrode were restored to the original uncoated state [214]. They also found that the neural recording function of the electrode with a thicker HG coating was remarkably lost. Hassarati et al. evaluated the performance of PEDOT/PVA hydrogel coatings for cochlear implants [215]. The results showed that the CSC values (124 mC/cm^2^) of coated electrodes were approximately 10-times larger than that (13 mC/cm^2^) of the Pt electrode. Due to the good flexibility of the PEDOT/PVA hydrogel coating, the coated electrode could restore to the original shape after bending.

For neural interfacing, the adhesion between the hydrogel coating and neural electrode is considered one of the most vital factors that determines the reliability and lifetime of the neural device. The delamination of hydrogel coatings from the neural electrode’s surface due to poor adhesion may lead to the failure of the neural electrode. Much research effort has been made to enhance the adhesion stability between the hydrogel coatings and the electrode substrate. Recently, strategies for achieving the strong adhesion of hydrogel coatings to the surface of neural electrodes by covalent bonding have been proposed. He et al. introduced a hybrid hydrogel coating composed of poly(ethylene glycol) diacrylate (PEGDA) and single-walled carbon nanotubes (SWNTs) [216]. In order to prepare the coating, a gold electrode was first modified with cysteamine (Cys), followed by Michael addition between Cys and PEGDA. The hybrid hydrogel coating was intact after 30 CV cycles. Furthermore, the hybrid hydrogel coating did not detach from the gold electrode after ultrasonication for 15 min, while the hydrogel coating without SWNTs detached after 10 s under ultrasonication. Moreover, the treatment of glass capillaries by 3-(trichlorosilyl) propyl methacrylate (TPM) for the improvement of the adhesion of PEG hydrogel was proposed by Spencer et al. (Figure 7c) [48]. Kleber et al. reported a method to produce conducting hydrogel attached to the surface of the conductive sites using 3-EBP silane [217]. The electrochemical stability of the conducting hydrogel was confirmed by a small decrease of the CSC (~1.9%) after 1000 CV cycles; no delamination could be observed visually for the coating after ultrasonication (Figure 7d,e). Although conducting hydrogel coatings can enhance the electrical and mechanical properties of neural microelectrodes, there are a few limitations for these hydrogel coatings regarding the capacity of facilitating neural regeneration to improve the integration of the electrode–nerve tissue interface. A new approach to embed neural cells into the hydrogel coatings of neural electrodes, called a “living electrode”, was proposed to improve the neuron survival rate and minimize the formation of scar tissues [218,219,220]. The feasibility, electrical and mechanical properties of the living electrode in vitro were comprehensively characterized by R. A. Green and coworkers [218]. In their work, the Pt electrode was first coated with a conducting hydrogel (CH) to improve its electrical properties, then a second hydrogel coating encapsulating the nerve cells was deposited on the CH-coated Pt electrode. The second hydrogel coating was degradable, and the rate of degradation was expected to match the extension and growth rates of the neural cells in order to effectively form an integrated tissue–electrode interface with indistinct borders between the synthetic device and the surrounding tissues.

A 3D printing technique has also been used for the construction of advanced microstructured hydrogels which are suitable for neural electrode coatings. The typical 3D printing methods of hydrogel include inkjet printing [221], light-based printing [222] and extrusion-based printing [223]. For example, Jiang et al. fabricated a three-dimensional collagen/silk fibroin scaffold which can support the adhesion, elongation and differentiation of neural stem cells in vitro, and can promote the repair of injured the spinal cords of rats in vivo [224]. In order to mimic the real neural tissue, Kuzmenko et al. 3D-printed conductive nanocellulose-based scaffolds for in vitro neural tissue growth and assessment. The cell culture studies demonstrated that, compared to the pure nanocellulose, cells cultured on the conductive guidelines printed by the nanocellulose-based ink exhibited better proliferation and differentiation, which were probably induced by the ink’s conductive property [225]. Rinoldi et al. designed a soft, biocompatible and conductive semi-IPN hydrogel which can improve the survival rates of neurons and astrocytes [222]. This hydrogel—as a 3D-printing ink—could be fabricated into micro-objects, which denotes a promising potential for novel neural tissue engineering applications. The resolutions of 3D printing technology vary from millimeters to submicrons. Thus, it is possible to precisely print the hydrogel coatings with specific morphologies and functions on the neural microelectrode by the 3D printing technique.

## 7. Conclusions

During the last few decades, various kinds of coating layers—which are designed to improve the electrical, mechanical and biological properties of the electrodes—have been used for neural interfacing. Lots of progress has been made, but many challenges—e.g., neural cell adhesion, neurite growth and chronic implantation—still exist in the way of pursuing a harmonious tissue–electrode interface. In order to improve the adhesion of neural cells on the surface of the coating, the surface microstructure and chemical composition of the coating can be modified to mimic the in vivo neural cells’ living environment. Selected topological features and surface modifications of bioactive molecules/growth factors can also lead to optimal neurite growth and neuron adhesion on the surface of the coating.

Another challenge is how to improve the long-term stability of the coating in vivo. During implantation, the electrochemical properties of the coating deteriorate due to degradation. The delamination of the coating from the neural electrode may happen after repeated stimulations. Moreover, the mechanical friction between the coating and tissue induced by the brain micromotion may result in the delamination of the coating. In order to enhance the mechanical stability of the coating on the neural electrode, the strategy of introducing covalent bonding between the coating and the neural electrode can be utilized.

From a biological perspective, the neural electrode would be considered to be a foreign object by the immune system; the function of the electrode can be dramatically hindered by acute protein fouling and chronic scar formation, which set an intensive insulating barrier between the electrode and the surrounding tissue. The barrier significantly elevates the physical distance between the electrode and the neural cells, and thus attenuates the signal transmission and causes signal loss along the pathway. In order to reduce the protein absorption, new advanced materials with anti-fouling and good biocompatibility can be introduced as coatings. Recently, zwitterionic materials have been studied as coatings for neural electrodes due to their superior resistance to nonspecific protein adsorption (less than 0.3 ng cm^−2^). Cui’s group grafted zwitterionic polymer poly(sulfobetaine methacrylate) (PSB) onto the silicon implant’s surface using a biomimetic catechol group [226]. The results of the in vivo tissue response tests showed fewer microglia and macrophages around the PSB-coated neural electrode compared to the bare electrodes. More importantly, fewer activated astrocytes were observed around the PSB-coated electrode compared to the control after 1 week of implantation, suggesting that the PSB coating may have the ability to enhance the duration of neural electrodes. Future research effort should aim to produce a tissue–electrode interface with high cell affinity. Multifunctional nanostructured composite coatings with excellent biomechanical and electrochemical properties, and capable of imitating the cell surface structure, will benefit the design of an ideal tissue–electrode interface.

## Data Availability

Data sharing not applicable.
